# Genome-wide comparative analysis of H3K4me3 profiles between diploid and allotetraploid cotton to refine genome annotation

**DOI:** 10.1038/s41598-017-09680-6

**Published:** 2017-08-22

**Authors:** Qi You, Xin Yi, Kang Zhang, Chunchao Wang, Xuelian Ma, Xueyan Zhang, Wenying Xu, Fuguang Li, Zhen Su

**Affiliations:** 1 State Key Laboratory of Plant Physiology and Biochemistry, College of Biological Sciences, China Agricultural University, Beijing, 100193 China; 2 State Key Laboratory of Cotton Biology, Institute of Cotton Research of Chinese Academy of Agriculture Sciences (ICR, CAAS), Anyang, Henan 455000 China

## Abstract

Polyploidy is a common evolutionary occurrence in plants. Recently, published genomes of allotetraploid *G. hirsutum* and its donors *G. arboreum* and *G. raimondii* make cotton an accessible polyploid model. This study used chromatin immunoprecipitation with high-throughput sequencing (ChIP-Seq) to investigate the genome-wide distribution of H3K4me3 in *G. arboreum* and *G. hirsutum*, and explore the conservation and variation of genome structures between diploid and allotetraploid cotton. Our results showed that H3K4me3 modifications were associated with active transcription in both cottons. The H3K4me3 histone markers appeared mainly in genic regions and were enriched around the transcription start sites (TSSs) of genes. We integrated the ChIP-seq data of H3K4me3 with RNA-seq and ESTs data to refine the genic structure annotation. There were 6,773 and 12,773 new transcripts discovered in *G. arboreum* and *G. hirsutum*, respectively. Furthermore, co-expression networks were linked with histone modification and modularized in an attempt to explain differential H3K4me3 enrichment correlated with changes in gene transcription during cotton development and evolution. Taken together, we have combined epigenomic and transcriptomic datasets to systematically discover functional genes and compare them between *G. arboreum* and *G. hirsutum*, which may be beneficial for studying diploid and allotetraploid plants with large genomes and complicated evolution.

## Introduction

Chromatin is an instructive DNA scaffold that can respond to external conditions and can be modulated through the modification of histones to change DNA accessibility and gene expression^1^. Notably, histone modifications as key epigenomic markers were thought to be correlated with gene expression both in terms of type and level^2, 3^. Essential processes, such as floral transition, cell differentiation, gametogenesis, and plant response/adaptation to environmental stresses, have been reported to be regulated by these histone markers^4^. In rice, several histone markers have been reported to be associated with transcriptional activation, such as di- and trimethylation of H3K4 (H3K4me2 and H3K4me3) and acylation of H3K9 and H3K27 (H3K9ac and H3K27ac). Among these, H3K4me3, a post translational modification (PTM) of histones^5, 6^, is known to be a euchromatic marker that may facilitate transcription initiation and elongation events and increase transcriptional regulation of genes^7, 8^. In plants, H3K4me3 histones are associated with transcription start sites (TSS) of expressed genes^9^ and are usually located from ∼150 bp upstream of the TSS to ∼500 bp downstream in rice^10^. Due to these characteristics, H3K4me3 has been widely used for plant biology research. In *Arabidopsis*, changes in H3K4me3 were associated with nonadditively expressed genes in allotetraploids^11^. In maize, high-stringency allele-specific H3K4me3 peaks discovered in endosperm are mostly co-localized with endosperm-specific maternally expressed genes (endo-MEGs)^12^. In cotton, H3K4me3 has been reported to define the expression bias of homologous genes in the allotetraploid cotton^13^.

Epigenomic knowledge has been widely used in functional genomics research, mostly in pre-transcriptional and transcriptional regulatory studies, to identify novel gene expression regulatory mechanisms. However, there are relatively few resources available to improve genomic structure annotation in plants. The genome-wide distribution of H3K4me3 has been profiled for chromatin study in plants^10, 14, 15^ and shows highly conserved profiling, peaking at the TSSs of genes and correlating with active transcripts^3, 16^. Therefore, H3K4me3 could be a marker for genome structure annotation. For example, H3K4me3, together with other histone modifications, is highly concurrent with transcript regions, which allowed the prediction of missing genes in rice gene annotation^6^. For non-coding genes, H3K4me3 was associated with transcription initiation as well and was integrated with expression profiles for primary transcription site identification of miRNAs in *Arabidopsis* ^17^. However, the effects of histone modification on the genomics and transcriptomics of multicellular organisms and on transcriptomic divergence between species are poorly understood^11^.

Cotton is a one of the most economically important crops worldwide due to the wide range of uses for its fibre and seeds. As a model polyploid plant, allotetraploid upland cotton *G. hirsutum* carries both A and D genome, making it an excellent model for heterosis research in crop domestication and improvement. Recently, genomes of polyploid cotton *G. hirsutum* and its donors, A sub-genome donor *G. arboreum* and D sub-genome donor *G. raimondii*, have been successfully sequenced. Available omics data increased explosively with the publication of the diploid and allotetraploid cotton genomes, which makes heterosis research at multi-dimensional levels (transcriptome and epigenome) more efficient in studying cotton’s evolution^18–21^. For instance, genome-wide histone modification caused expression bias during the evolution of polyploid cotton^13^. Based on previous works, diploid *G. arboreum* has a genome size that is nearly twice as large as *G. raimondii*, and allotetraploid cotton *G. hirsutum* has a more complex genome. In addition, the current annotated version of the genome assembly has no alternative splicing patterns and no non-coding sequences, so there might be some improvements to be made for gene structure refinement and novel gene discovery.

Here, DNA fragments associated with H3K4me3 modification have been successfully sequenced in the root tissue of *G. hirsutum*, as well as the root and stem tissues of cotton *G. arboreum*. According to whole genome-wide mapping, H3K4me3 is conserved across different species of cotton, but the enrichment ratio of genic regions was quite low compared to model plants. Therefore, we first tried to combine epigenomic data with other information, such as RNA-seq and expression sequence tags (ESTs), to help refine known gene annotations. Taking full advantage of ChIP-seq data and public RNA-seq data, 6,773 and 12,773 new transcripts were discovered in *G. arboreum* and *G. hirsutum*, respectively, including coding and non-coding transcripts. In addition, differential H3K4me3 modifications correlated with changes in transcript levels were explored to better understand gene transcription and regulation. This work may help experimental scientists to discover more functional genes in cotton and apply them for agricultural molecular breeding. Finally, we developed a platform named MOAP (http://structuralbiology.cau.edu.cn/MOAP/) for novel transcript browsing and searching.

## Results

### Common pattern of tri-methylation of histone H3 lysine 4 (H3K4me3) in cotton

**Figure 1:**
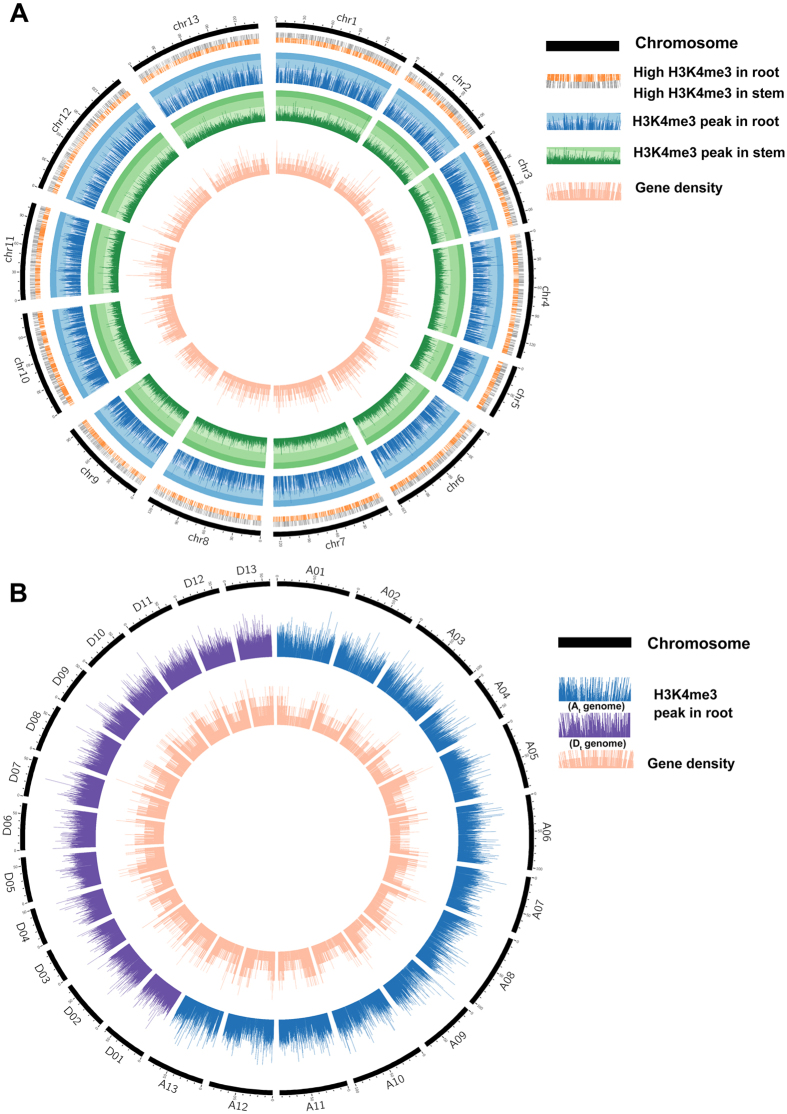
Genome-wide distribution of H3K4me3 and gene density. (**A**) The histone modification peaks of H3K4me3 in root (blue) and stem (green), and gene density (pink) distribution along the whole *G. arboreum* genome. In the calculation of gene density, 10,000 bp is used as a unit. The peak height indicates the enrichment score of H3K4me3. Histone modification peaks in orange and grey are root-up H3K4me3 peaks and stem-up H3K4me3 peaks, respectively. The differential histone modification peaks were identified by the software MACS, which compared H3K4me3 histone modification density between root and stem. (**B**) The histone modification peaks of H3K4me3 in root and gene density (pink) distribution among the whole *G. hirsutum* genome. The peaks in blue are in the At genome (donor was *G. arboreum*) and peaks in purple are in the Dt genome (donor was *G. raimondii*). The unit of gene density is 10,000 bp.

According to the quality control, the mapped ratios of three H3K4me3 histone ChIP-seq samples (root and stem in *G. arboreum*, root in *G. hirsutum*) were all above 90%, which indicates that the data should be reliable (Table 1). First, total peaks of histone modification deposition and the number of these peaks related to different genomic regions were calculated (Table 1). We found that the number of H3K4me3 peaks were conserved in different tissues of *G. arboreum*. In addition, the number of peaks in allotetraploid cotton was about twice as much as in diploid cotton. Based on a comparison between the distribution pattern of H3K4me3 and gene density, H3K4me3 was found more frequently in high gene density regions at the whole-genome level (Fig. 1). Taking chromosome A07 for example, there were more H3K4me3 peaks on the chromosome ends, and the gene densities of these regions were high as well (Fig. 1B).

**Table 1:** The statistics of reads mapped and peak prediction in H3K4me3 ChIP-seq data among two tissues and species in cotton.

H3K4me3 samples	Peaks	Filtered reads	Mapped ratio
*G. hirsutum* root	77128	22502102	95.58%
*G. arboreum* root	39054	28232174	92.85%
*G. arboreum* stem	38568	29584862	96.94%

**Figure 2:**
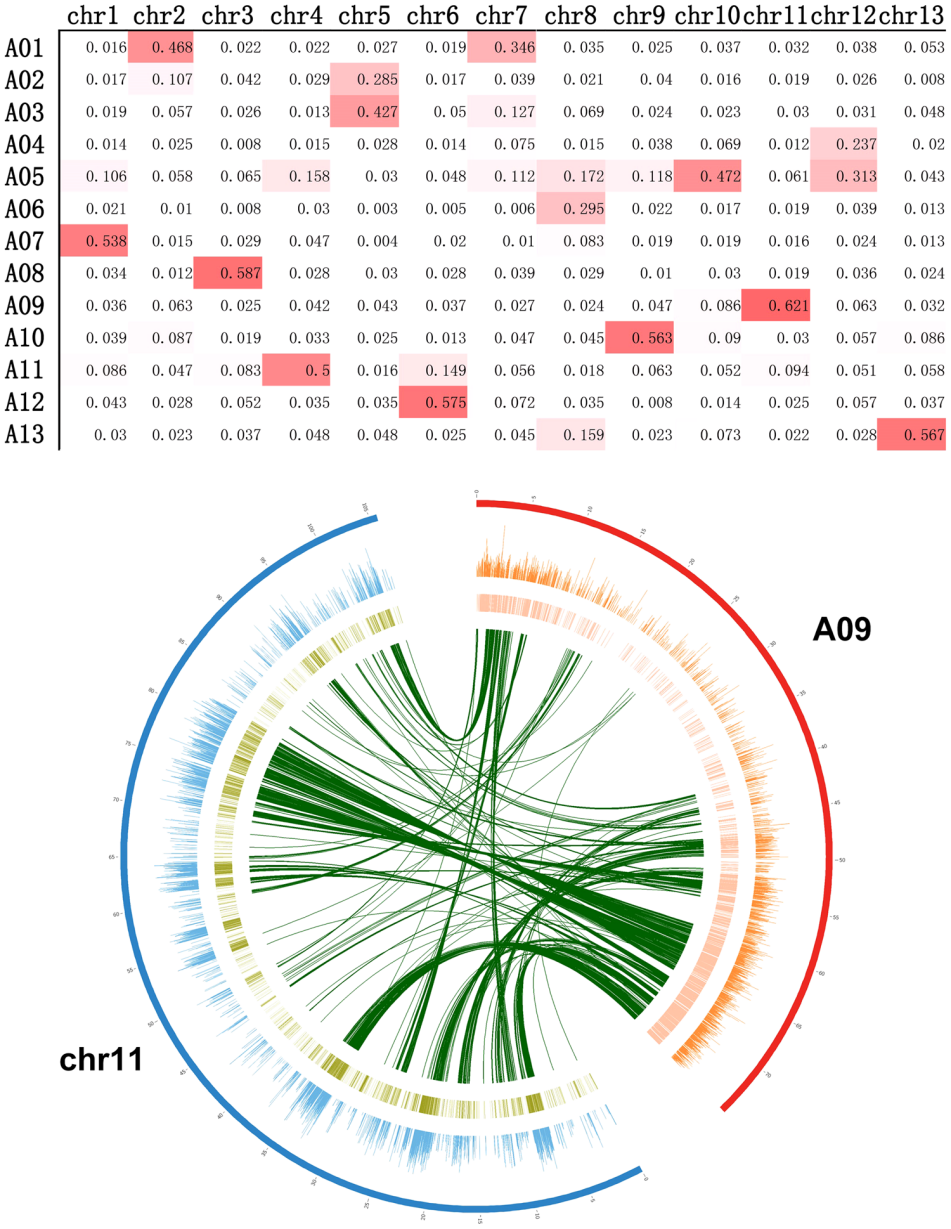
Conservation between diploid and allotetraploid cotton. (**A**) The red or white boxes stand for the ratio of orthologous gene pairs between each chromosome from *G. arboreum* (chr1-chr13) and *G. hirsutum* (A01-A13). (**B**) A comparison of H3K4me3, gene expression and orthologue between each pair of chromosomes. Data are displayed with circos software using four layers: the outer circle stands for the A09 (red) and chr11 (blue) chromosome, the second circle stands for H3K4me3 modification peak distribution along A09 (orange) and chr11 (blue), the third circle stands for gene expression density (high expression indicated by a dark colour) among A09 (pink) and chr11 (green), and the curves of the inner layer link orthologous genes in the two chromosomes.

After analysing whole-genome trends, H3K4me3 modification was compared between orthologous gene pairs to elucidate the deposition pattern of this histone marker during cotton evolution. As *G. arboreum* was the ancestral donor of the A sub-genome (At genome) in *G. hirsutum*, we mainly calculated orthologous gene pairs between the whole genome in *G. arboreum* (chr1-chr13) and the At genome in allotetraploid cotton *G. hirsutum* (A01-A13). A heatmap was drawn to compare the orthologue ratios between the ancestral and modern cotton (Fig. 2A). Based on the distribution of all orthologue ratios, most ratios were less than 0.05. However, each chromosome in *G. arboreum* had a relatively high ratio (close to 50%) with a chromosome in *G. hirsutum*. Colinearity was not obvious with the A-progenitor genome (*G. arboreum*), and gene loss was most likely an ongoing process in allotetraploid cotton^21^. Therefore, we defined ratios close to 50% or over 50% as ‘high conservation’. Apart from 4 chromosomes (chr5, chr7, chr8 and chr12), most chromosomes in *G. arboreum* were highly conserved with their receptors, as they could be linked to a chromosome of *G. hirsutum* sharing about half of the orthologous gene pairs. Among them, the chromosome pair A09 and chr11 were the most highly conserved and were selected to study the comparison between H3K4me3 and transcript expression at the chromosome level. The circos figure clearly described conserved correlation among transcript profiles, histone modification profiles and evolution. For instance, highly expressed active transcripts usually had high levels of the H3K4me3 modification near them in the donor chromosome (chr11), and their orthologues were also highly expressed and were surrounded by enriched histone modification in the A09 chromosome of *G. hirsutum* (Fig. 2B). This result supports the conservation between orthologous gene pairs at the epigenomic level, which also suggested that H3K4me3 is a conserved histone marker and is correlated with active transcripts during cotton evolution similar to the model plant^3, 16^.

### Association of H3K4me3 and gene transcriptional levels

**Figure 3:**
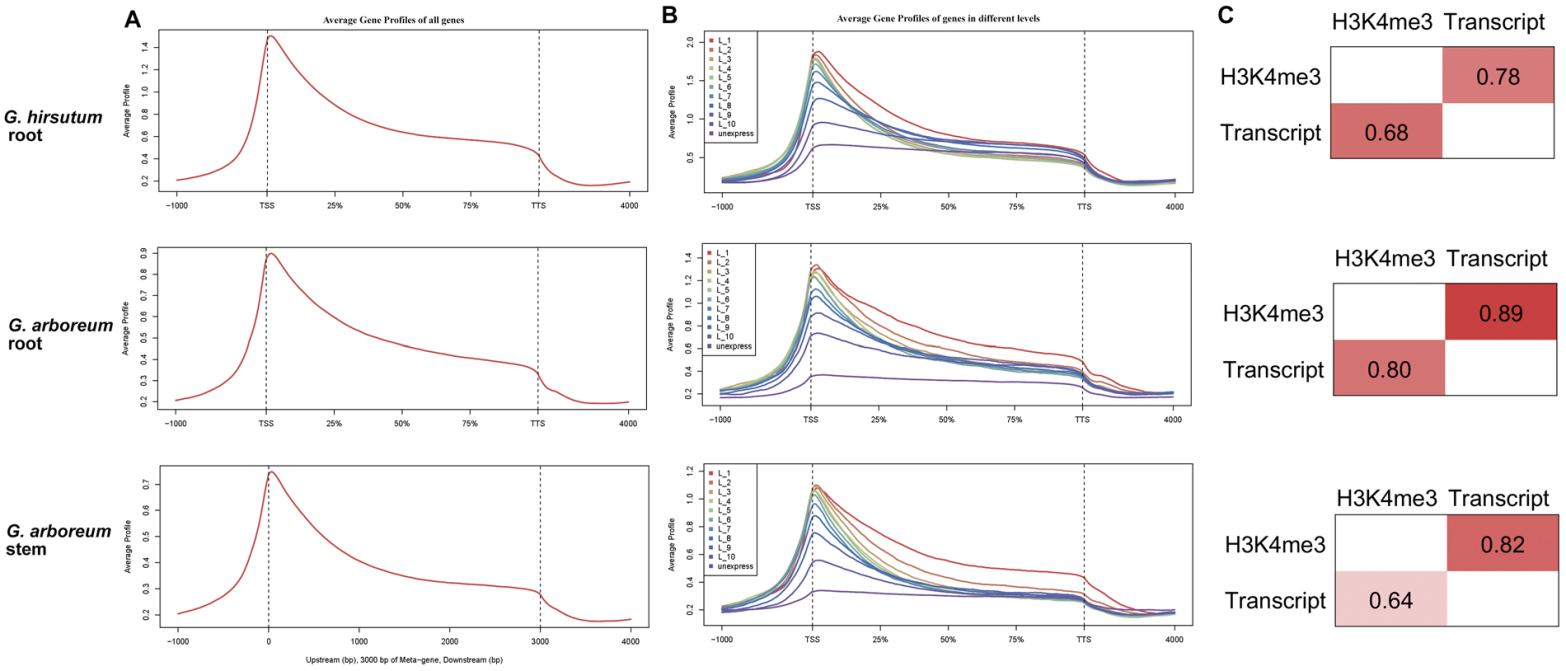
H3K4me3 associated with gene structure and expression. (**A**) The average gene profiles of H3K4me3 in cotton root and stem were generated from CEAS software using normalized sequencing read density. The gene body was divided into three equal parts to standardize different gene lengths, and the 1 kb upstream and downstream regions were also included for profile making. (**B**) The average gene profiles of H3K4me3 among different expression levels. Genes are divided into 11 groups according to their expression (FPKM value). The genes having FPKM > 0 are divided into 10 equal parts and the remaining genes which have FPKM = 0 are gathered and tagged as “unexpressed”. L1 is the highest-level group and is marked in red. L10 is the lowest level and is marked in dark blue. (**C**) The relationship between histone modification and active transcript expression. The numbers are standard for concurrence frequency computation for H3K4me3 histone modifications and active transcripts. For example, 0.78 means that 78% active transcripts contain the H3K4me3 modification in *G. hirsutum* root.

As a transcriptionally active epigenetic marker, the H3K4me3 modification has been correlated with changes in transcript levels in model plants. Here, we classified all transcripts into 10 groups based on their expression profiles in root and stem tissues of two cotton species to detect relationships between H3K4me3 modification and gene transcriptional regulation. The average profiles of the ten groups showed the following characteristics: (1) the pattern of average H3K4me3 profiles were conserved either in different tissues (root and stem) and/or in different species (*G. arboreum* and *G. hirsutum*; Fig. 3A); (2) H3K4me3 accumulated mainly near TSSs of active transcripts and was enriched downstream of TSSs (Fig. 3A); and (3) genes with high expression values in root were correlated with high histone modification (Fig. 3B). In the polyploid cotton, 78% of the active transcripts had H3K4me3 modification near their TSSs, and 68% of the H3K4me3 modifications accumulated near active transcripts in root tissue. In root and stem tissues of diploid cotton, 89% and 82% of active transcripts had H3K4me3 nearby, and 80% and 64% histone modification were associated with active transcripts, respectively (Fig. 3C). Based on the three kinds of average profiles, H3K4me3 modification peaked downstream of TSSs in cotton, which was consistent with the pattern in other plants like *Arabidopsis* (Fig. 3A). Therefore, the H3K4me3 histone modification is positively correlated with active transcripts in cotton.

**Figure 4:**
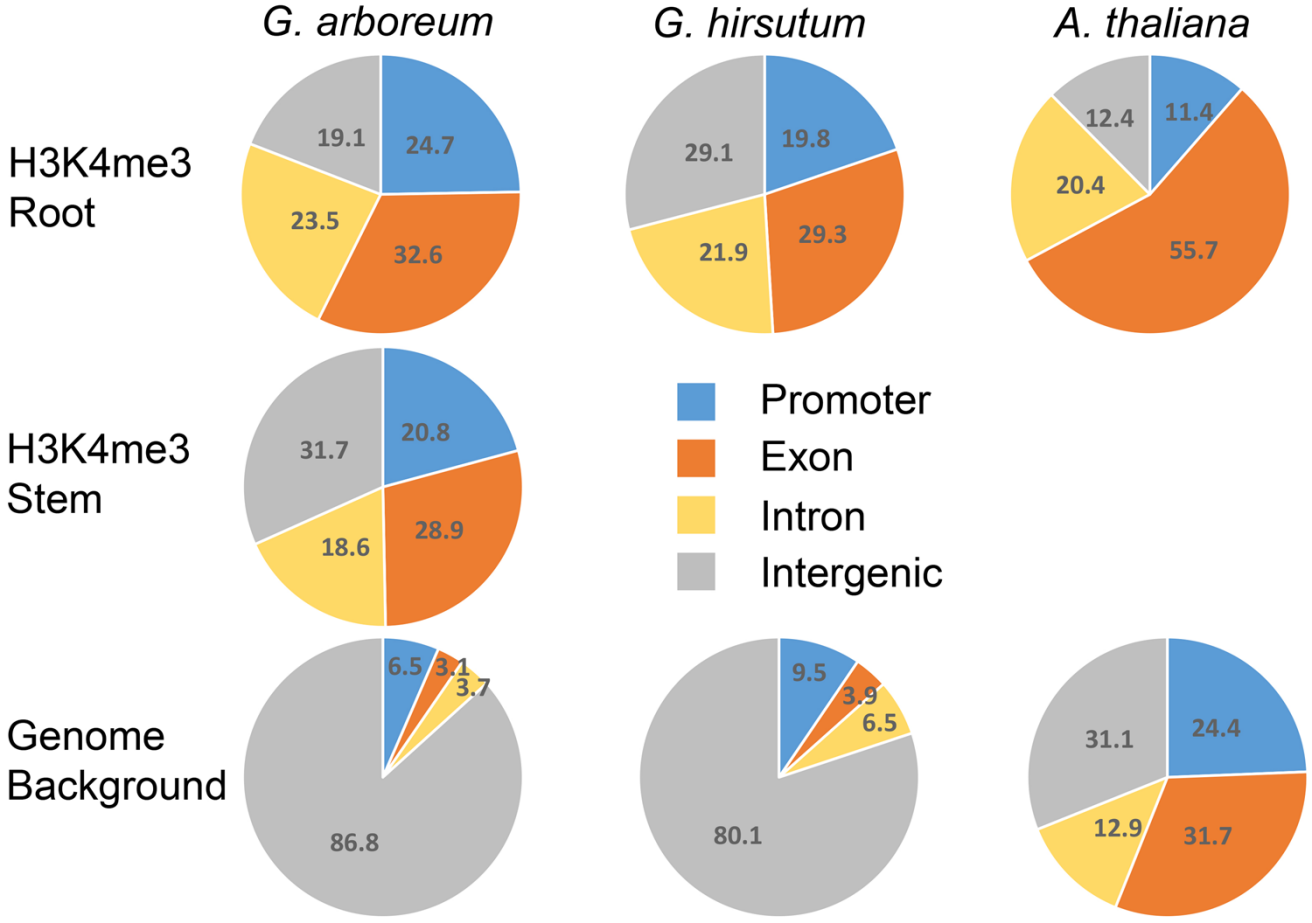
Genome background and H3K4me3 distribution ratio. The distribution of H3K4me3 histone modification and genome background within 4 main gene regions of known genes in root and stem tissues of three plant genomes (*G. arboreum*, *G. hirsutum* and *Arabidopsis thaliana*).

Due to the large genome of cotton, four genomic regions, including promoter, exon, intron and intergenic, were classified for comparison of H3K4me3 distribution among different tissues and species (Fig. 4). The pie graphs of genomic regions had both similarities and differences in H3K4me3 peak distribution. For the similarities, the histone marker was mainly enriched in genic regions (promoter, exon and intron) in both cotton and *Arabidopsis*. For the differences, the ratio of H3K4me3 in each genomic region showed species-specific and tissue-specific patterns. For example, 55.7% of H3K4me3 peaks were deposited in the exon region of *Arabidopsis* root, which was about twice as much as in *G. hirsutum*. In *G. arboreum* stem, 31.7% of H3K4me3 peaks were deposited in intergenic regions, which was approximately 1.7 times as much as in the root. These differences may be caused by the different genomic structure of the three species. In *Arabidopsis*, 68.9% of the genomic region has been annotated, whereas there were only 13.2% and 19.9% annotation of gene regions in *G. arboreum* and *G. hirsutum*, respectively, suggesting that the annotation of cotton was limited and still has much room for improvement.

### Identification of novel transcripts in annotated cotton genome with epigenomic maps

**Figure 5:**
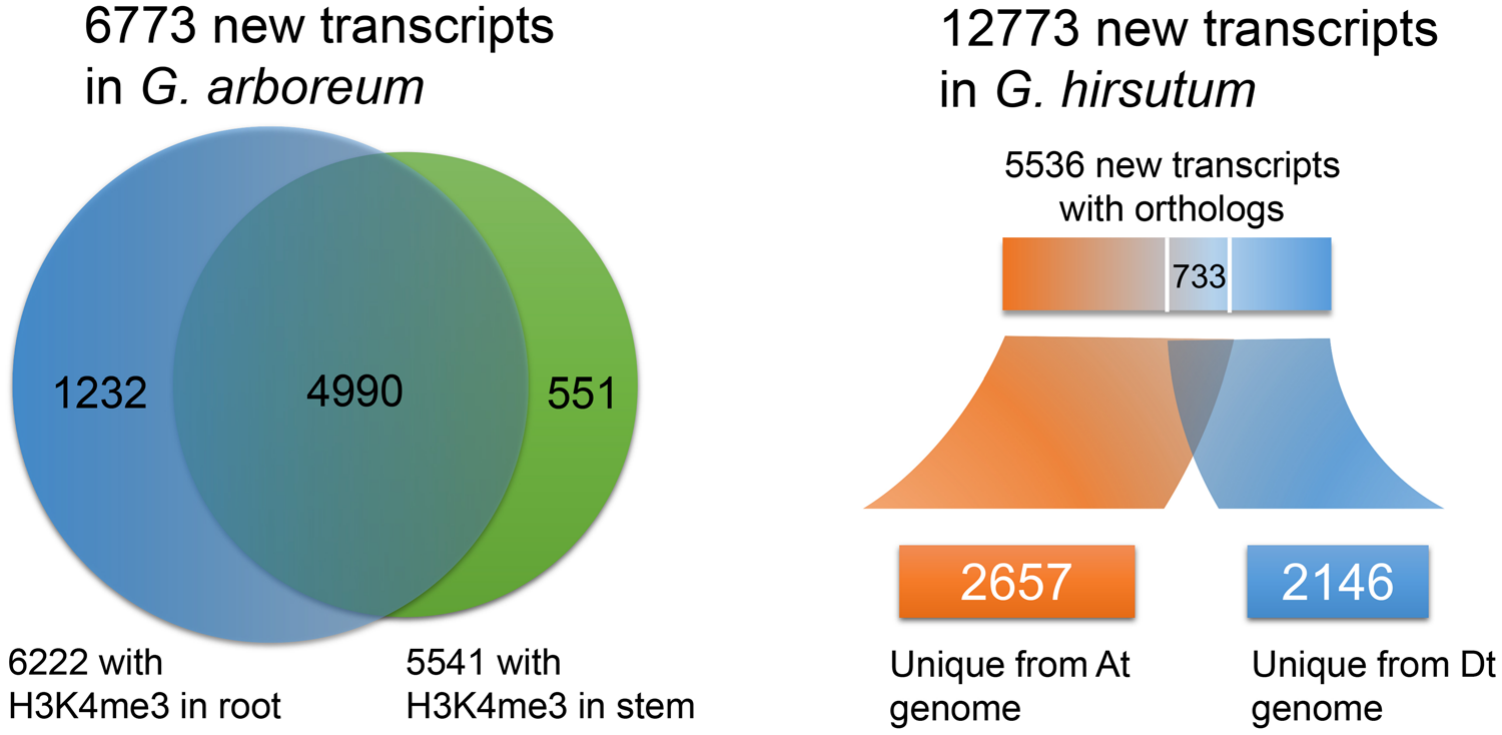
Detailed comparison for novel transcripts. (**A**) New transcripts, totalling 6,773, from *G. arboreum* derived from RNA-seq data, and new transcripts that have H3K4me3 evidence in root and stem. (**B**) The distribution of 5,536 new transcripts of *G. hirsutum* with orthologues in donor genomes. 2,657 transcripts only have an orthologue in the At genome donor (*G. arboreum*) and 2,146 transcripts only have an orthologue in the Dt genome donor (*G. raimondii*).

**Figure 6:**
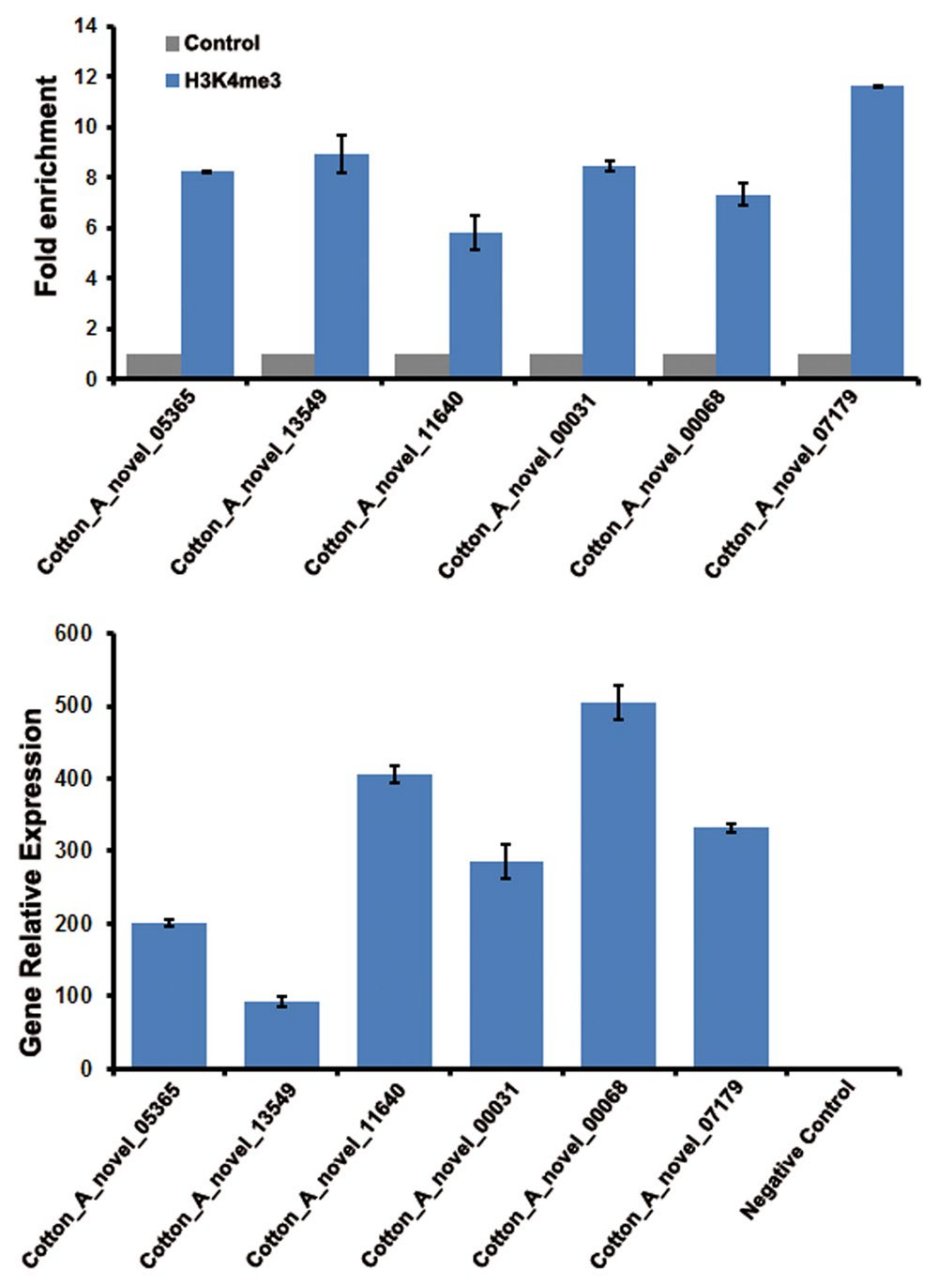
Results of qRT-PCR validation. The qRT-PCR confirming results of six selected novel transcripts which were Cotton_A_novel_05365, Cotton_A_novel_13549, Cotton_A_novel_11640, Cotton_A_novel_00031, Cotton_A_novel_00068 and Cotton_A_novel_07179.

**Figure 7:**
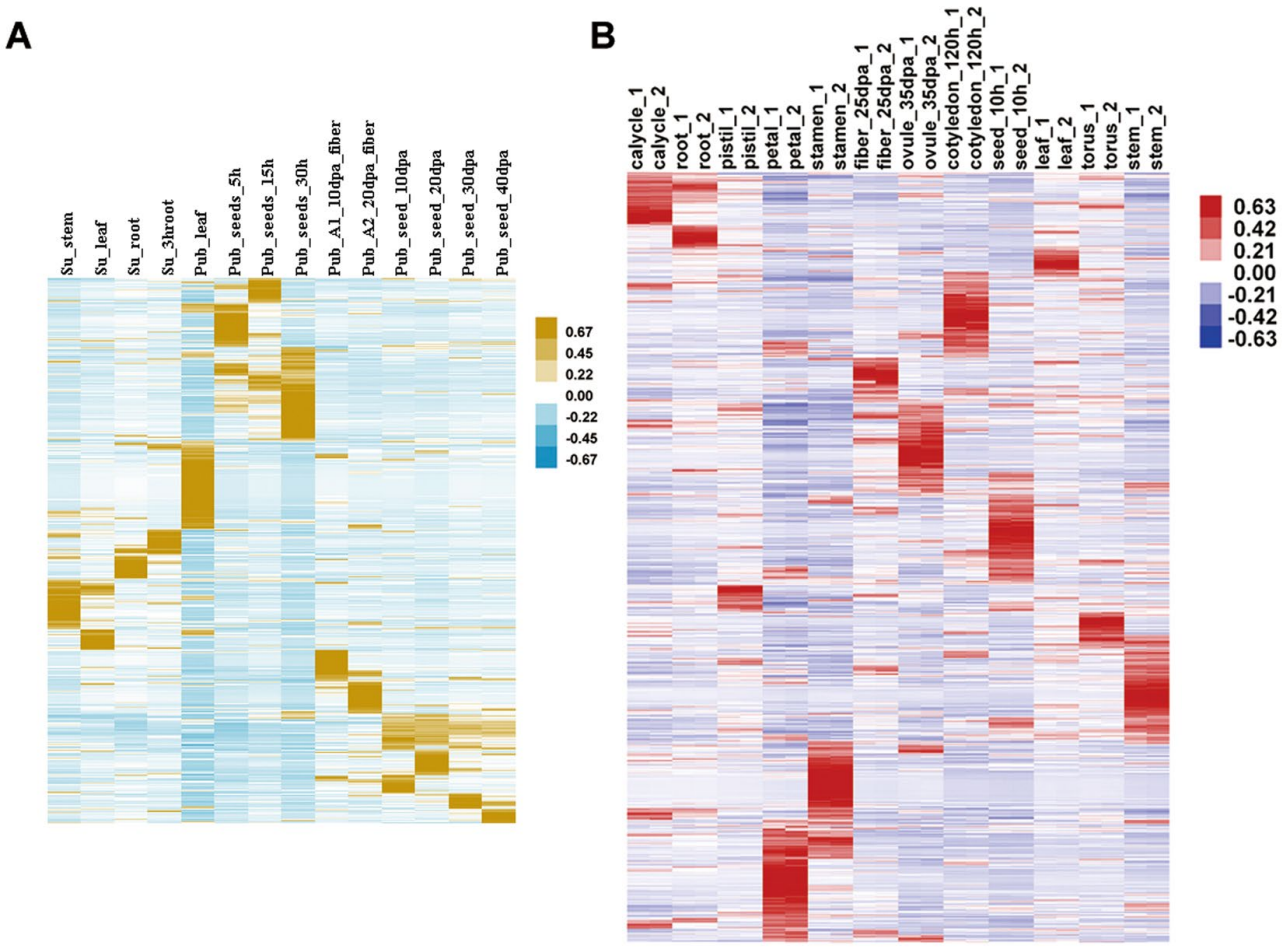
Differential expression situation of novel transcripts. (**A**) Heatmap clustering of newly identified transcripts in *G. arboreum* among 14 expression profiles of six different tissues, including stem, root and leaf, fibre, seed and seedlings The “Su” stands for in-house data, and the “Pub” stands for public data. Each coloured box indicates the z-score. (**B**) Heatmap clustering of newly identified transcripts in *G. hirsutum* among 24 expression profiles from twelve different tissues, including torus, calycle, pistil, stamen, petal, leaf, stem, root, seed, cotyledon, ovule and fibre. Each box indicates the z-score.

Based on the epigenomic maps of the two cotton species, we were aware of the limitations of genomic annotation. Due to the strong relationship between H3K4me3 and transcript levels, it was possible and necessary to refine the current gene annotation. Previous studies had used groups of RNA-seq data to build gene models^22–26^ and identify potential exon regions^19^ in a plant genome. According to average profiles, we regarded H3K4me3 as a marker for the gene strand. Therefore, ChIP-seq and RNA-seq data were combined to investigate new transcripts in cotton, including their structure and putative function. Here, we integrated our self-coded scripts with some published toolkits to design a workflow for new transcript identification in plant species with a reference genome (Fig. S2). First, the transcriptomics data was used for possible novel transcript discovery by TopHat for reads mapping and Cufflinks for new transcript model calculation^27^. After that, the cuffmerge package was used to merge transcripts predicted from RNA-seq samples from multiple developmental stages. Because most of the candidates were non-directional, the locations of H3K4me3 peaks were added for TSSs identification of the novel transcripts. Specifically, we manually classified the transcript body into three equal parts: left body region, middle body region and right body region. When a correlated H3K4me3 peak (when the distance between the centre of the transcript and the H3K4me3 peak was less than 2000 bp) was upstream of the TSS or in the left body region, the strand of the associated transcript was defined as “+”; when the peak was inside the middle body region, the strand of the transcript was defined as “middle”; and when the peak was inside or downstream of the right body region, the strand of the transcript was defined as “−”. For the efficiency of new gene discovery, all cotton expression sequence tags (ESTs) were obtained from CottonGen^28^ for sequence strand prediction and verification. Finally, the novel transcripts were compared to known cotton ESTs for linking nearby exons and confirming the sequence strand (Fig. S2). For the diploid cotton *G. arboreum*, we analysed 14 expression profiles containing multiple developmental stages for novel transcript identification (Table S1), two H3K4me3 profiles (in stem and root) for sequence strand selection, and ESTs for confirmation. After filtering, we obtained 6,773 novel transcripts, and 4,990 of them correlated with the H3K4me3 histone marker in root and stem tissues (Fig. 5A; Table S3, S5). The UCSC Genome Browser showed the gene structure of six selected novel genes and the corresponding expression pattern in all RNA-seq and ChIP-seq data. We noticed that H3K4me3 was enriched in genic regions and showed the highest peaks near the TSS (Fig. S3). The qRT-PCR results also showed the accuracy of the predicted novel transcripts (Fig. 6; Table S2). To annotate these transcripts, we used the Coding Potential Calculator^29^ online service to help predict the protein coding capability of those sequences. Additionally, we used BLAST against multiple data sources, including CDS sequences of *Gossypium raimondii* and *Gossypium hirsutum*, UniprotKB database, Rfam and known miRNA precursors of cotton species. Finally, we defined 5,111 putative protein-coding transcripts and 1662 non-coding transcripts. Among these, there were 1,613 putative new ncRNAs sequences (Table S3). We recalculated the FPKM value of each novel transcript in all RNA-seq expression profiles and then clustered novel transcripts based on the FPKM value. The heatmap result suggested these novel transcripts were usually expressed in specific tissues (Fig. 7A).

**Figure 8:**
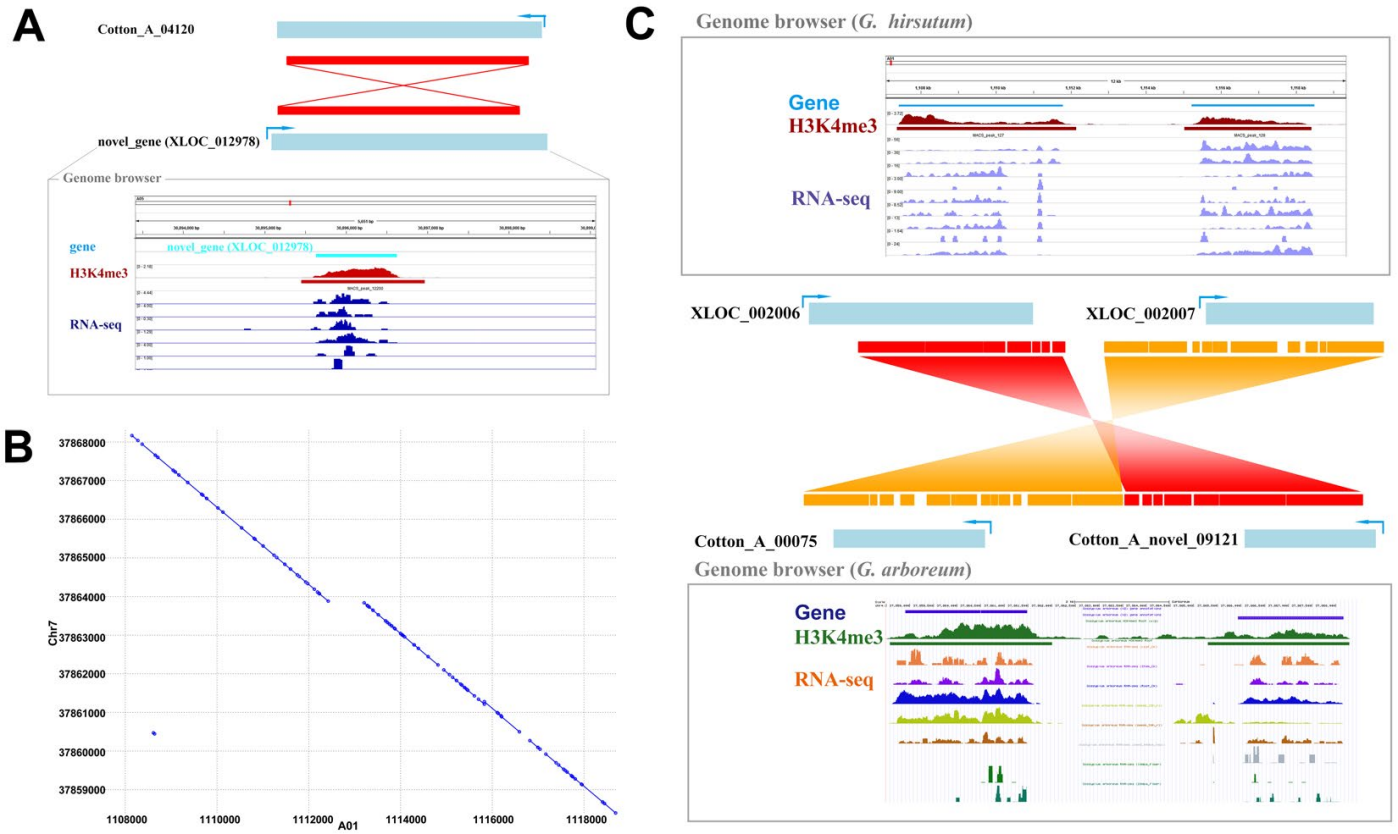
Synteny analysis between *G. hirsutum* and *G. arboretum*. (**A**) The Blastn result between the *G. arboreum* gene Cotton_A_04120 and a novel transcript in *G. hirsutum* XLOC_012978. The red rectangles indicate alignment regions with high scores. The lower panel presents profiles of H3K4me3 and RNA-seq surrounding XLOC_012978 in SVG software. (**B**) Detail of the reverse synteny regions between chromosome Chr7 (37,868,000 to 37,859,000) in *G. arboreum* and A01 (1,108,000 to 1,118,000) in *G. hirsutum*. (**C**) Detail of the known genes and novel transcripts in the (**B**) synteny region. XLOC_002006 and XLOC_002007 are novel transcripts in *G. hirsutum*. The novel transcript Cotton_A_novel_09121 in *G. arboreum* shares a reverse collinearity relationship with XLOC_002006. The known gene Cotton_A_00075 in *G. arboreum* shares a reverse collinearity relationship with XLOC_002007.

Following the same workflow, 12,773 novel transcripts (Table S3, S6) were predicted by 24 expression profiles (Table S1) and a root H3K4me3 profile in *G. hirsutum* (Fig. 5B). Based on the systemic annotation, 7,330 putative protein-coding transcripts and 5,443 non-coding transcripts were defined. The heatmap clustering results also showed tissue-specific expression (Fig. 7B). According to a previous report, putative gene-loss events may have occurred during genome duplication and fusion, and there were 228 transcripts in the A genome (*G. arboreum*) and 141 transcripts in the D genome (*G. raimondii*) that have been lost in the tetraploid cotton *G. hirsutum* ^21^. Because of conservation in cotton evolution, high synteny regions between novel transcripts in *G. hirsutum* and known genes in *G. arboreum* can increase the reliability of novel transcript prediction. Here, 21 of the lost A-genome genes and 22 of the lost D-genome transcripts were retrieved^21^, which indicated the limitations of cotton genic annotation (Table S4). Based on sequence alignment (e-value = 0, length coverage = 87.7%), we found that the novel transcript XLOC_012978 might be orthologous to the reported lost A-genome gene Cotton_A_04120 (Fig. 8A)^21^. In addition, the genomes of *G. arboreum* and *G. hirsutum* were compared to verify the novel transcripts from polyploid cotton at the synteny level. As a result, there were two gene pairs in a selected high synteny region of the two genomes (Fig. 8B). One pair was novel transcript XLOC_002006 in *G. hirsutum* and novel transcript Cotton_A_novel_09121 in *G. arboreum*, the other was novel transcript XLOC_002007 and known gene Cotton_A_00075 (Fig. 8C).

### Modularized analysis of differential H3K4me3 modifications with changes in transcript levels

The pattern of H3K4me3 is conserved at the whole-genome level in cotton. However, there were also many active transcripts with differential histone modification.

#### Differential H3K4me3 modifications during cotton evolution

**Figure 9:**
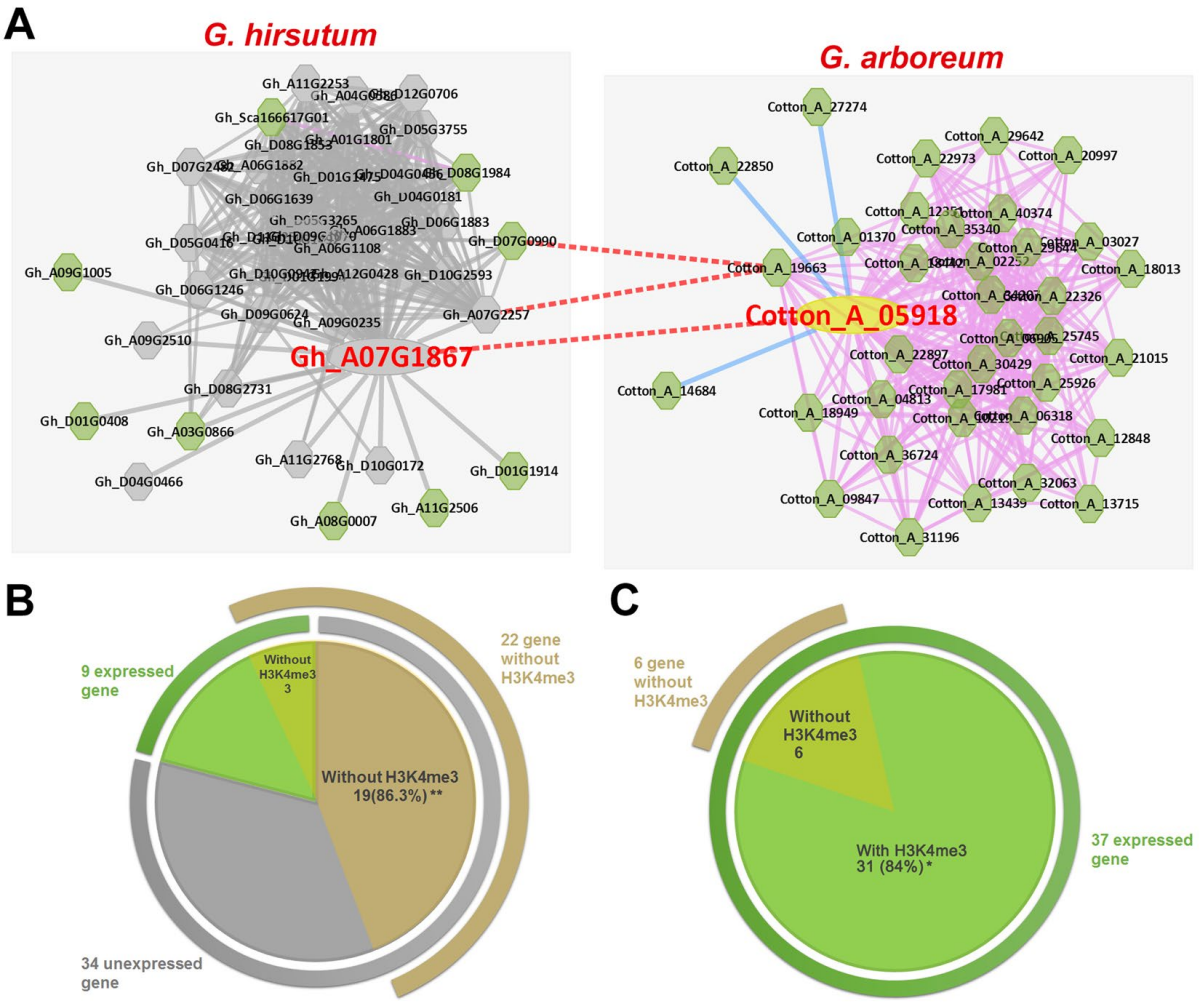
Modularized comparison of differential histone modification during evolution. (**A**) The co-expression networks of the orthologous gene pair of Gh_A07G1867 and Cotton_A_05918 are compared. Grey and green nodes are unexpressed and expressed genes in root tissues, respectively. A pink or blue line links positive co-expressed or negative co-expressed gene pairs, respectively. A red dotted line links orthologous gene pairs. (**B**) There are 43 co-expressed genes in the root-preferential co-expression network of Gh_A07G1867, including 9 expressed genes (green loop covers) and 34 unexpressed genes (grey loop covers). Among them, 22 co-expressed genes lack the H3K4me3 histone modification. In *G. hirsutum*, 50,113 genes had the H3K4me3 modification and 20,365 genes lacked it. In the sub-network, 34 genes were unexpressed in root, and among them, 15 genes had the H3K4me3 and 19 genes did not. Fisher’s Exact Test was performed, and p-values of 0.001 are marked by **. (**C**) There are 37 co-expressed genes in the root-preferential co-expression network of Cotton_A_05918, and all of them are expressed in root. Among these, 6 co-expressed genes did not have H3K4me3 histone modification. In *G. arboreum*, 28,923 genes had the H3K4me3 modification and 12,408 genes did not. In the sub-network, 37 genes were expressed in root and among them, 31 genes had the H3K4me3 and 6 genes did not. Fisher’s Exact Test was performed, and the p-value was 0.073 and marked by *.

Orthologous gene pairs generally function in similar biological processes, but there were expression-related changes in a subset of orthologous gene pairs, which may have occurred during cotton evolution. The causes of this situation are probably complex. Fortunately, the data on H3K4me3 histone modification of root tissues in diploid *G. arboreum* and polyploid *G. hirsutum* enabled us to analysis the causes at the epigenome level. According to the ccNET platform, 96,466 orthologous pairs were established through a bidirectional BLAST algorithm-based alignment and strict E-value cutoff (1E-55)^30^. The gene expression view tool set FPKM 0.24 and FPKM 0.17 as cutoffs to identify whether the gene was expressed in *G. arboreum* and *G. hirsutum* ^30^. After comparison, 4,600 orthologous pairs showed root-preferential expression in diploid cotton but were not expressed in polyploid root. Co-expression network analysis was used to modularly detect relationships between differential gene expression and histone modification. For example, the root-preferential co-expression networks of orthologue gene pair (Gh_A07G1867 and Cotton_A_05918) were significantly different, as all co-expressed genes were expressed in *G. arboreum* root, whereas 80% of the co-expressed genes were not expressed in *G. hirsutum* root (Fig. 9A). When considering the epigenomic results, 84% (P < 0.07, Fisher’s Exact Test in method) of the expressed genes were associated with H3K4me3 in *G. arboreum* (Fig. 9B), whereas 86.3% (P < 0.001, Fisher’s Exact Test in method) of genes without H3K4me3 were not expressed in *G. hirsutum* (Fig. 9C). Thus, the high ratio values both confirm that H3K4me3 is positively correlated with active genes in cotton, and suggest a co-functional relationship of the epigenome and transcriptome in gene regulation.

#### Differential H3K4me3 modifications between root and stem

**Figure 10:**
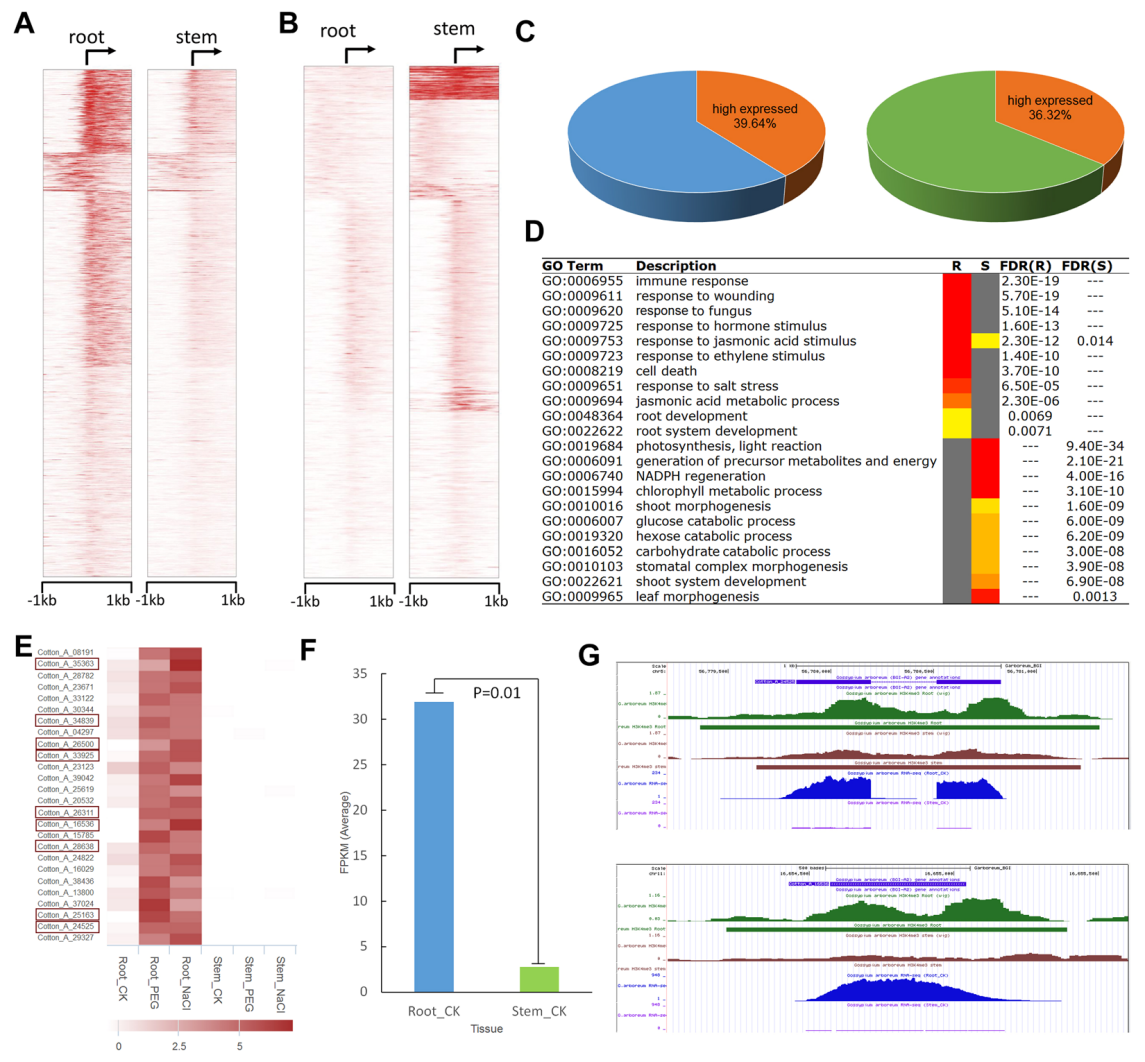
Modularized comparison of differential histone modification during development. Heatmaps for H3K4me3 around the TSSs of 4,871 root-up modification (**A**) and 1,608 stem-up modification (**B**) genes in *G. arboreum*. K-means were used to cluster them into four groups. For each gene, the histone modification intensities are displayed along –1-kb to 1-kb regions around the TSSs. The colour represents H3K4me3 levels. (**C**) 39.64% of the 4,871 root-up modification genes are highly expressed in root (left) and 36.32% of the 1,608 stem-up modification genes are highly expressed in stem (right). (**D**) GO enrichment results for genes having both high H3K4me3 modification and expression in root (R) and stem (S) are selected here. The red and grey boxes with “GO: 0006955” stand for root-up genes that are enriched significantly, whereas stem-up genes are not significant. (**E**) A heatmap of 26 gene expression profiles in root and stem. All the genes shown are involved in the co-expression network of Cotton_A_24525 (GaGSTU7). Genes highlighted with red rectangles have root-up H3K4me3 modification. (**F**) The average FPKM value of the 9 co-expression genes highlighted in (**E**) are compared to control root and stem tissues. (**G**) The H3K4me3 profiles in UCSC genome browser. Gene Cotton_A_24525 and its co-expressed gene Cotton_A_16536 with root-up modification are presented.

In diploid cotton *G. arboreum*, 4,871 transcripts correlated with more H3K4me3 modification in root (Fig. 10A), and 1,608 transcripts correlated with more H3K4me3 modification in stem (Figs 1A and 10B). Therefore, we further examined the effects of epigenomics on gene regulation. Approximately 40% of genes showed consistent correlations between epigenomic state and transcriptomic level. For example, 39.64% of the 4,871 transcripts with high levels of the H3K4me3 modification in root were expressed highly in root, and 36.32% of the 1,608 transcripts with high H3K4me3 accumulation in stem also expressed highly in stem (Fig. 10C). Gene Ontology (GO) analysis showed that root-up genes were mainly enriched in root development and response to biotic and abiotic (salt) stress, which could indicate that *G. arboreum* has water stress tolerance, or that because the root is directly exposed to the growth medium with PEG or salt, the response genes were more active in root^31, 32^. The stem-up genes were more enriched in light regulation, leaf and shoot morphogenesis and carbohydrate catabolic processes, which involve in the main functions of stem and leaf ^33^ (Fig. 10D). In addition, a co-expression network was applied for modularized analysis. Among the genes with high H3K4me3 modification in root, Cotton_A_24525, a member of Glutathione S-Transferase Gene Family (GaGSTU7), was reported to have a high expression level in root^34^. Based on ccNET platform analysis^30^, genes that are co-expressed with GaGSTU7 were also expressed significantly highly in root (Fig. 10F), and 34.6% (9/26) genes were associated with more H3K4me3 modification in root (Fig. 10E,G). Here, transcriptional regulators (TFs) with differential H3K4me3 were analysed to determine possible affected processes (Fig. S4A). After clustering, we found they were related to tissue-specific expression. For instance, there were more members of the AP2 family in the group with high H3K4me3 modification in root (Fig. S4A), and these TFs were also highly expressed in root (Fig. S4B). Network-modularized analysis of one AP2 member Cotton_A_05798, an orthologue of BABY BOOM (AT5G17430), which is mainly expressed in embryos and lateral root primordium^35^, showed the module was root preferential selection, because half of the co-expressed genes were not expressed in stem (Fig. S4D). Moreover, there were 36.7% (11/30) positive co-expressed genes with root-up histone modification (Fig. S4C). Finally, the statistical analysis suggested that differential H3K4me3 correlated with changes in transcript level may both contribute to affect gene regulation among tissues.

## Discussion

In this study, we performed ChIP experiments targeting H3K4me3, generated the epigenomic profiles and compared them between tissues (root and stem) and species in cotton (*G. arboreum* and *G. hirsutum*). From the results, we found that H3K4me3 mainly occurred in genic regions and was enriched around TSSs, which was highly consistent with other plants that had epigenomic maps already (Fig. 3A). In addition, this histone marker was confirmed to be correlated with transcriptional activation in cotton (Fig. 3B,C). However, comparing epigenomic maps between two cotton species and the well-studied model plant *Arabidopsis*, we found that the distribution ratio of the genic regions (promoter, exon and intron) was quite low in cotton, which suggested that the current genomic annotation has significant limitations (Fig. 4). Therefore, a feasible protocol was designed to discover novel transcripts by integrating epigenome and transcriptome data (Fig. S2). As a result, 6,773 and 12,773 novel transcripts were identified in diploid and polyploid cotton, respectively. The qRT-PCR and synteny comparison improved the accuracy of the predicted results. For instance, the lost A-genome gene Cotton_A_04120 reported previously^21^ retrieved the orthologous gene XLOC_012978 in novel transcripts of *G. hirsutum* (Fig. 8A). In the high synteny region between two cotton genomes (Fig. 8B), two novel transcripts in *G. hirsutum* retrieved a defined gene, Cotton_A_00075, and a novel gene, Cotton_A_novel_09121, as orthologous genes in *G. arboreum* (Fig. 8C).

Despite the conserved pattern of the H3K4me3 profile, differential histone modifications could possibly affect gene regulation as well^36^. The modularized analysis of differential H3K4me3 modifications indicated the efficiency of integrating the epigenome and transcriptome, such as in the case of genes with tissue-preferential H3K4me3 modifications and significant enrichment in some tissue-related biological processes (Fig. 10D). Genes in a co-expression network showed differential histone modification correlated with changes in transcript level (Figs 10E, S4C), and different frequencies of TF families with differential H3K4me3 modification levels were clustered in tissue-specific expression groups, suggesting a dynamic regulation mechanism in tissues (Fig. S4A,B). In addition, clustering analysis on orthologue gene pairs not only confirmed that H3K4me3 modification correlated with active genes but also suggests co-function of the epigenome and transcriptome in gene regulation during cotton evolution (Figs 2B and 9A,B).

There were shortcomings and needed improvements in refining cotton genomic annotation. First, the length of H3K4me3 peaks was sometimes too long to define the novel transcript strand. Methods like ESTs and manual correction were integrated, but a fraction of novel transcript strands were still unclear. Therefore, profiles of promoter-related markers like RNA polymerase II (Pol II), DNase I hypersensitive (DH) sites, and cap analysis of gene expression (CAGE) will be needed to improve and confirm novel transcript structure in cotton^17^. Moreover, incorporating histone markers associated with transcriptional repression into the analysis, such as H3K27me3 or H3K9me2, will make transcript strand or tissue-specific expression patterns more convincing. Additionally, histone modification profiles after stress treatments are lacking. For example, the BBM gene Cotton_A_05798 and its co-expressed genes exhibited a positive relationship between gene expression and H3K4me3 modification. However, expression clustering results showed that they were down-regulated after PEG treatment. Threrefore, we need histone modification profiles with stress treatments to understand the mechanism of cotton adaptability to different environments^37^.

## Conclusion

In summary, we elucidated conserved H3K4me3 distribution patterns of cotton, applied the results to refine genomic structure annotation (Fig. S5), and supplied a modularized comparison strategy to analyse the conservation and diversity of gene function during cotton development and evolution. We believed that modularized integration of the epigenome and transcriptome will be helpful for complex cotton genome analysis and further contribute to cotton molecular breeding and cotton production improvement. This procedure is also a successful attempt for plant biology process detection, especially in polyploid species with large genome sizes and limited annotation.

## Materials and Methods

### Plant Materials and Growth Conditions

Cotton (*G. arboreum* L. cv. Shixiya, *G. hirsutum* L. cv. TM-1) seeds were immersed in water for 1 d at 30 °C and allowed to germinate on sterilized soil in plates maintained under the following conditions: 28/25 °C, 12/12 h of light/darkness, and relative humidity of 80%. After 4 days, properly germinated seedlings were transferred to black plastic tanks filled with a nutrient solution^32^ and allowed to grow until they had produced 4–6 leaves. The tissues from stem and root were harvested for experiments.

### Chromatin Immunoprecipitation

Chromatin immunoprecipitation (ChIP) was performed as described previously with minor modification^38^. Approximately 15 g of root and stem tissues were homogenized to a fine powder in liquid nitrogen and re-suspended in TBS (10 mM Tris, pH 7.5, 3 mM CaCl_2_, 2 mM MgCl_2_, 0.1 mM PMSF, and 2/5 of a tablet of complete mini protease inhibitor cocktail (Roche Applied Science, Indianapolis, USA) with 0.5% Tween 40. The nuclei were purified on a sucrose gradient and digested by micrococcal nuclease (MNase: Sigma, St Louis). The nucleosome samples were first incubated with pre-immune rabbit serum (1:100 dilution) and then with 4% protein A Sepharose (GH healthcare Bio-Sciences AB, Uppsala) for 4 h, after which they were centrifuged. The histone modification antibodies were same as those used in our previous work^6^. The supernatant was incubated with anti-trimethyl-histone H3 (Lys 4; H3K4me3, Millipore 07–473, Temecula) at 4 °C overnight. An equal amount of pre-immune rabbit serum, which served as a nonspecific binding control in each ChIP experiment, was used in the control experiments. Then, the samples were incubated with 25% protein A Sepharose at 4 °C for 2 h. After centrifugation, the pellet (bound) fractions were subjected to a series of washes, and the immune complexes were eluted from the washed beads using elution buffer. Immunoprecipitated DNA was extracted using phenol/chloroform and precipitated with ethanol.

Two biological replicates treated with antibodies (treatment) and two replicates with a mock treatment (control) were used in each ChIP experiment. ChIP sequencing experiments were undertaken only when two treatment samples both showed significant differences compared to the control samples after quantitative real-time PCR. Quantitative real-time PCR analysis of ChIPed DNA was performed on the Applied Biosystems 7500 Real Time PCR System to determine the relative fold enrichment of modified histone-associated sequences in the bound fractions. The gene-specific primers were same as those used in the relative gene expression experiments. Relative fold enrichment (RFE) was calculated as 2^–ΔΔCT^ ± standard deviation (SD), where ΔΔCT = ΔCT (positive control) − ΔCT (negative control). The two treatment replicates were then mixed and sent for sequencing.

To validate the reliability of ChIP antibodies, we performed a ChIP-qPCR experiment, and the results (Fig. S1) showed high ChIP efficiency for H3K4me3.

### Isolation of RNA and Quantitative Real Time PCR

The cotton samples were homogenized in liquid nitrogen before RNA isolation. Total RNA was isolated using a modified CTAB method and purified using Qiagen RNeasy columns (Qiagen, Hilden, Germany).

Reverse transcription was performed using an M-MLV kit (Invitrogen). The samples, 10 µl each containing 2 µg of total RNA and 20 pmol of random hexamers (Invitrogen), were maintained at 70 °C for 10 min to denature the RNA and then chilled on ice for 2 min. The reaction buffer and M-MLV enzyme (20 µl of the mixture contained 500 µM dNTPs, 50 mM Tris-HCl (pH 8.3), 75 mM KCl, 3 mM MgCl_2_, 5 mM dithiothreitol, 200 units of M-MLV, and 20 pmol random hexamers) was added to the chilled samples, and the samples were maintained at 37 °C for 1 h. The cDNA samples were diluted to 8 ng/µl for QRT-PCR analysis.

For quantitative real-time PCR, assays were performed in triplicate on 1 µl of each cDNA dilution using the SYBR Green Master Mix with an ABI 7500 sequence detection system as prescribed in the manufacturer’s protocol (Applied Biosystems). The gene-specific primers were designed using PRIMER3 (http://primer3.ut.ee/). The amplification of 18S rRNA was used as an internal control to normalize all data (forward primer, 5′-CGGCTACCACATCCAAGGAA-3′; reverse primer, 5′-TGTCACTACCTCCCCGTGTCA-3′). The gene-specific primers are listed in Table S4. The relative quantification method (ΔΔCT) was used for quantitative evaluation of the variation between replicates.

### Data Source

Cotton root and stem ChIP-seq data and four samples of mRNA-seq data from three tissues (root, leaf and stem)^39^ were sequenced using IlluminaHiSeq™ 2000 followed by standard protocols. Other mRNA-seq data for *G. arboreum* and *G. hirsutum* were downloaded from the Sequence Read Archive (SRA; Table S1). *Aradibopsis* ChIP-seq data (GSE50636) was downloaded from the NCBI Gene Expression Omnibus (GEO)^40^. The first published genome of *G. arboreum* was used as a reference genome^19^, the *G. hirsutum* genome referred to the Nanjing Agricultural University version^21^, and the *Arabidopsis* genome referred to the release 10 (TAIR10) version^41^.

### Analysis of ChIP-seq data

ChIP-seq reads of histone modifications were mapped to the genome of cultivated cotton *G. arboreum* using the Bowtie (v2.1.0) short read aligner. The MACS (v1.4.1) program was used for peak calling (default parameters). The CEAS (v1.0.2) software was used to analyse characteristics of peak distribution across genomic regions. The genomic region was classified in a pie chart generated by CEAS: 5′UTRs and 3′UTRs were classified as Exons, whereas downstream sequences was classified as Intergenic regions. The circular ring represents epigenomic profiles and gene density along all chromosomes in cotton and was displayed using the Circos localized program. Data on histone modifications were normalized using MACS output files and displayed on a customized UCSC Genome Browser.

In *G. arboreum*, the differential histone modification peaks between root and stem were calculated by the MACS program. The parameter -t/–treatment was set as the H3K4me3 profiling of root, and the parameter -c/–control was set as the H3K4me3 profiling of stem. Therefore, positive peaks indicate there was more H3K4me3 histone modification in root than that in stem, whereas negative peaks indicate the opposite.

### Analysis of RNA-seq data

To identify novel transcripts, in-house and public mRNA-seq reads were mapped to the reference genome using Tophat (v2.0.9) software. Cufflinks (v2.2.0) was used to calculate the expression level of each transcript using default parameters. Cluster (v3.0) software was used to do hierarchical analysis using tissue-specific genes screened by a z-score test, and the results were displayed by Java Treeview (v1.1.6r4).

### An integrative approach to identify novel transcripts

First, Tophat, Cufflinks and Cuffmerge were utilized in order to predict putative novel transcripts using 14 samples and 24 samples of mRNA-seq data of *G. arboreum* and *G. hirsutum*, respectively. Second, H3K4me3 peaks were predicted by Model-based analysis using ChIP-Seq (MACS). Third, the histone modification peaks with peak centres near the novel transcripts (<2000 bp) were used to identify the transcript strand. When the centre was close to the left end of a novel transcript, then the strand was forward; when the centre was near the right end, then the strand was reverse, and when the centre was near the middle part, then the strand was defined as “middle”. H3K4me3 peaks from stem and root tissues were used independently. Fourth, EST location sites download from CottonGen^28^ were applied for transcript merging and sequence strand proofing semi-manually.

### Annotation of newly identified transcripts and synteny analysis

We used three methods to determine the coding ability of new transcripts, which were Blast against cDNA sequences of *G. arboreum*, *G. raimondii* and *G. hirsutum* using the Blastn (v2.2.30+) program (E-value < 1 × 10^−5^), Blast searches were also performed against the protein sequences from the UniprotKB database using the Blastx (v2.2.30+) program (E-value < 1 × 10^−3^) and online Coding Potential Calculator (v0.9r2) prediction.

The core program ‘mumer’ of software MUMmer (v3.23) was used to align the entire genomes of *G. arboreum* and *G. hirsutum* by default parameters.

### Fisher’s Exact Test

In *G. hirsutum*, 50,113 genes had the H3K4me3 modification and 20,365 genes lacked the H3K4me3 modification. In the sub-network of *G. hirsutum*, 34 genes were not expressed in root and among these, 15 genes had the H3K4me3 modification and 19 genes lacked it. The p-value was 0.001 (Fig. 9B). In *G. arboreum*, 28,923 genes had the H3K4me3 modification and 12,408 genes did not. In the sub-network of *G. arboreum*, 37 genes were expressed in root, and among these, 31 had the H3K4me3 and 6 genes did not. The p-value for this set was 0.073 (Fig. 9C).

## Electronic supplementary material

Supplementary file

Table S3

Table S4

Table S5

Table S6
