# Supplementary material for: Genome-wide comparative analysis of H3K4me3 profiles between diploid and allotetraploid cotton to refine genome annotation
Source: Sci Rep. 2017 Aug 22;7:9098. doi: 10.1038/s41598-017-09680-6 (PMC5567255; doi:10.1038/s41598-017-09680-6)
Supplement: Supplementary file 1 — Supplementary file [file 41598_2017_9680_MOESM1_ESM.pdf]

Genome-wide comparative analysis of H3K4me3 profiles between diploid and allotetraploid cotton to refine genome annotation

Qi You†[1], Xin Yi†[1], Kang Zhang†[1], Chunchao Wang[1], Xuelian Ma[1], Xueyan Zhang[2], Wenying Xu[1], Fuguang Li*[2], Zhen Su*[1]

[1]State key Laboratory of Plant Physiology and Biochemistry, College of Biological Sciences, China Agricultural University, Beijing, 100193, China
[2]State Key Laboratory of Cotton Biology, Institute of Cotton Research, Chinese Academy of Agriculture Sciences (CAAS), Anyang, Henan 455000, China

† These authors contributed equally to this work

* Authors for correspondence
Zhen Su
e-mail: zhensu@cau.edu.cn; fax: +86-10-62731380

Fuguang Li
e-mail: aylifug@hotmail.com; fax: +86-372-2562256

Supplementary figures and tables

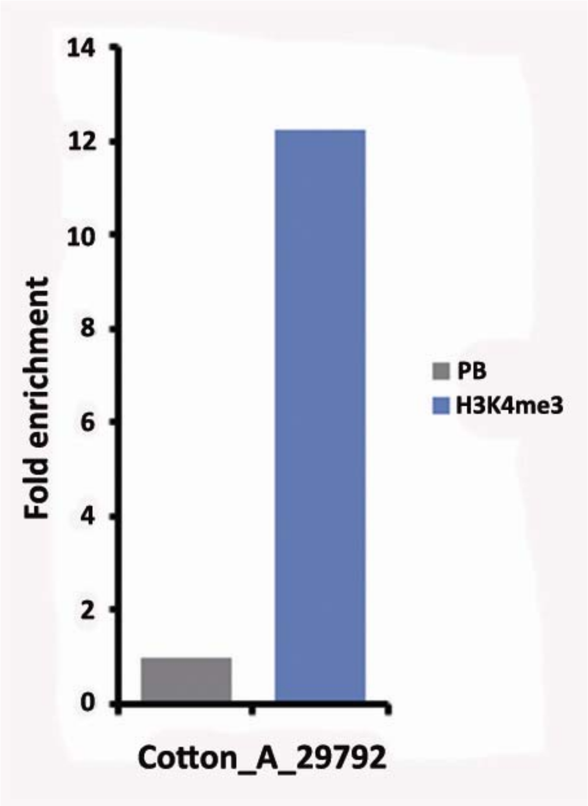

Fig. S1. Results of ChIP–qPCR validation.
Primers are designed using the sequence of the annotated *G. arboreum* gene Cotton_A_29792 (EUKARYOTIC ELONGATION FACTOR 5A-1) which has an enriched H3K4me3 peak.

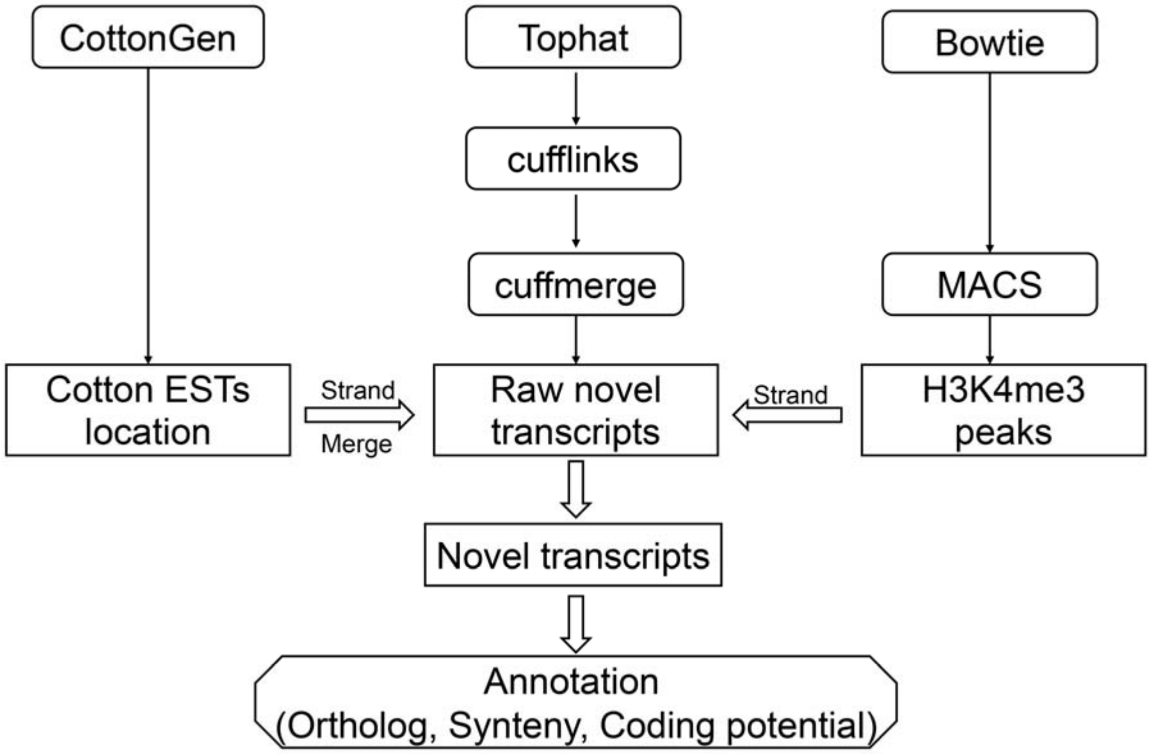

Fig. S2. Protocol for novel transcript identification.

An integrative method for epigenomics profiling and new transcript identification of diploid cotton *G. arboreum* and polyploid cotton *G. hirsutum*. The details are described in the methods section.

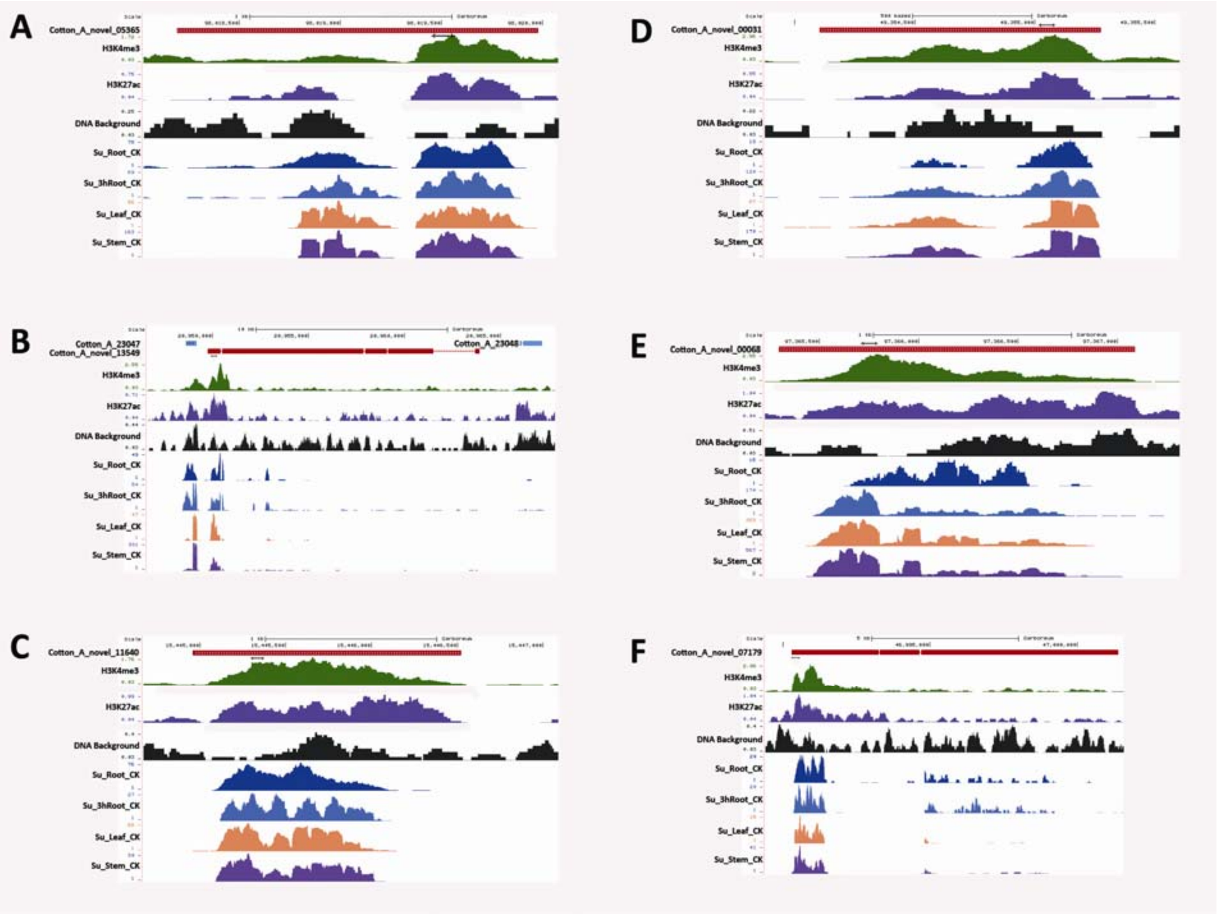

Fig. S3. Profiles of six selected transcripts.

Six screen-shots of the UCSC Genome Browser showing the epigenomic maps of six novel genes were selected from the newly identified transcripts. The first track shows the 5′ and 3′ primer sequences we used in the RT-PCR experiments. The second track shows the new transcript locations. The third track is the H3K4me3 histone modification situation in wig format. The last four tracks show the RNA-seq expression level with our own in-house data. (**A**) The UCSC Genome Browser screen-shot of Cotton_A_novel_05365. (**B**) The UCSC Genome Browser screen-shot of Cotton_A_novel_13549. (**C**) The UCSC Genome Browser screen-shot of Cotton_A_novel_11640. (**D**) The UCSC Genome Browser screen-shot of Cotton_A_novel_00031. (**E**) The UCSC Genome Browser screen-shot of Cotton_A_novel_00068. (**F**) The UCSC Genome Browser screen-shot of Cotton_A_novel_07179.

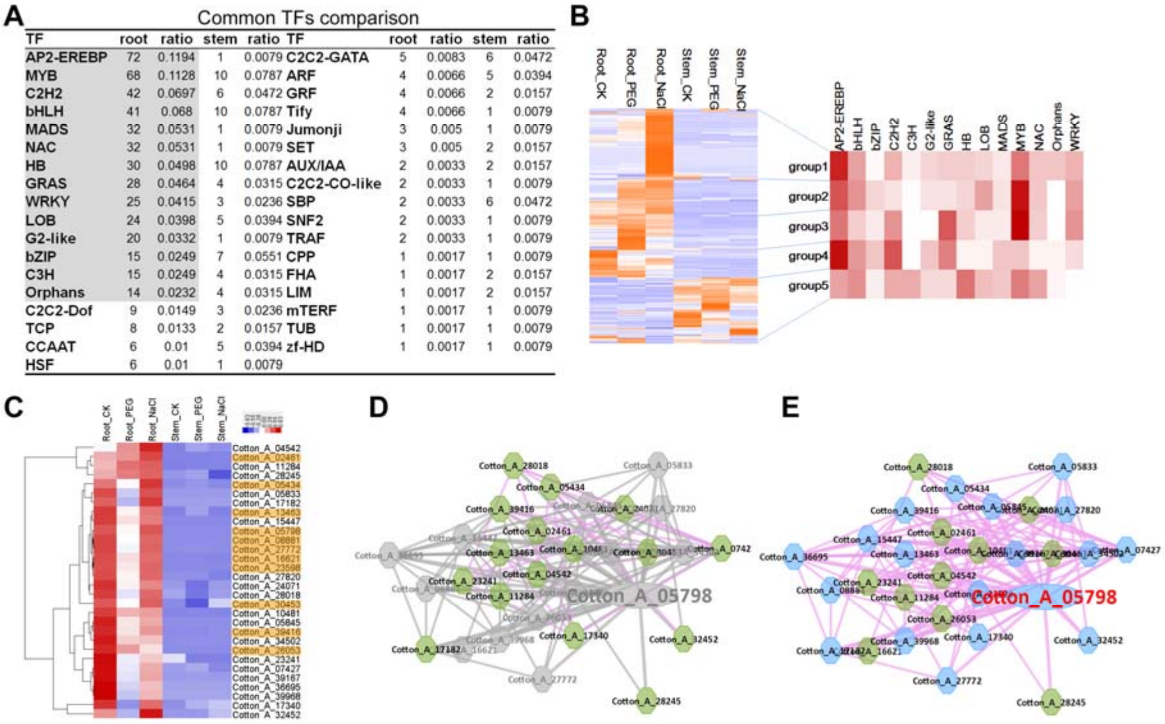

A Common TFs comparison

TF	root	ratio	stem	ratio	TF	root	ratio	stem	ratio
AP2-EREBP	72	0.1194	1	0.0079	C2C2-GATA	5	0.0083	6	0.0472
MYB	68	0.1128	10	0.0787	ARF	4	0.0066	5	0.0394
C2H2	42	0.0697	6	0.0472	GRF	4	0.0066	2	0.0157
bHLH	41	0.068	10	0.0787	Tify	4	0.0066	1	0.0079
MADS	32	0.0531	1	0.0079	Jumonji	3	0.005	1	0.0079
NAC	32	0.0531	1	0.0079	SET	3	0.005	2	0.0157
HB	30	0.0498	10	0.0787	AUX/IAA	2	0.0033	2	0.0157
GRAS	28	0.0464	4	0.0315	C2C2-CO-like	2	0.0033	1	0.0079
WRKY	25	0.0415	3	0.0236	SBP	2	0.0033	6	0.0472
LOB	24	0.0398	5	0.0394	SNF2	2	0.0033	1	0.0079
G2-like	20	0.0332	1	0.0079	TRAF	2	0.0033	1	0.0079
bZIP	15	0.0249	7	0.0551	CPP	1	0.0017	1	0.0079
C3H	15	0.0249	4	0.0315	FHA	1	0.0017	2	0.0157
Orphans	14	0.0232	4	0.0315	LIM	1	0.0017	2	0.0157
C2C2-Dof	9	0.0149	3	0.0236	mTERF	1	0.0017	1	0.0079
TCP	8	0.0133	2	0.0157	TUB	1	0.0017	1	0.0079
CCAAT	6	0.01	5	0.0394	zf-HD	1	0.0017	1	0.0079
HSF	6	0.01	1	0.0079					

Fig. S4. Modularized comparison of differential histone modification between tissues.

(A) The table displays 603 TFs with root-up modification and 127 TFs with stem-up modification. The columns, titled 'root' or 'stem' summarize the total members in the corresponding TF families. The ratio column shows the distribution of each TF family (e.g., 0.1194=72/603). (B) The left heatmap shows the TFs highlighted in (A) clustered into 5 groups based on their expression profiles. The right heatmap exhibits the distribution of TF families in each group. Specifically, AP2 family members appear most and bZIP family members appear least in group1. (C) A clustered expression heatmap of 30 genes in the positive co-expression network of Cotton_A_05798. A total of 11 genes highlighted in orange have root-up H3K4me3 modification. (D-E) The expression view result of 30 genes in the positive co-expression network of Cotton_A_05798. A pink line links positive co-expression gene pairs. Grey and green nodes are unexpressed and expressed genes in stem tissue (D). Blue nodes are genes that were down-regulated in root tissue after PEG treatment (E).

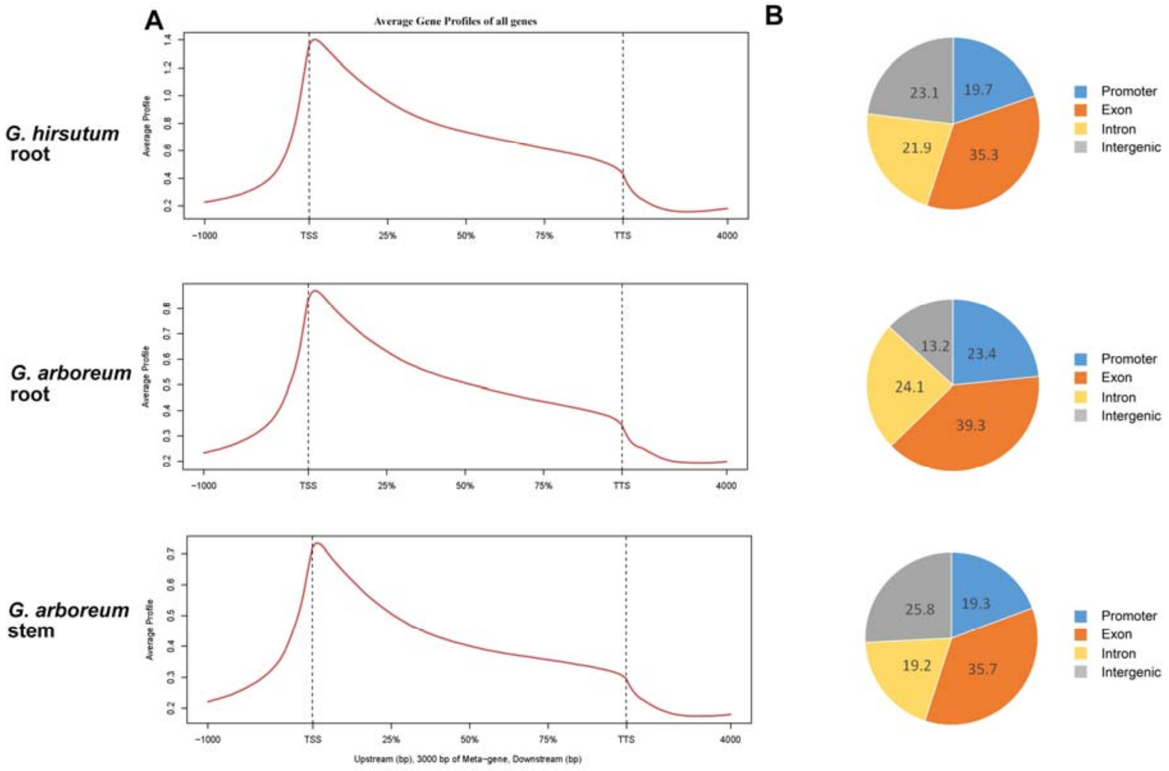

Fig. S5. **H3K4me3 distribution in the newest genome annotation.**

(A) The average novel and known gene profiles of H3K4me3 in cotton root and stem were generated from CEAS software using normalized sequencing read density. The gene body was divided into three equal parts to standardize different gene lengths, and the 1 kb upstream and 4 kb downstream region were also included for profile generation. (B) The distribution of H3K4me3 histone modification within 4 main gene regions of known and novel genes in root and stem tissues of *G. arboreum* and root tissue of *G. hirsutum*.

Table S1. Detailed information of RNA-seq data of cotton.

Samples	Reference
**Detailed information of RNA-seq data of *G.arboreum***	
Su_Stem_CK(Pair-end)<br>Su_Root_CK(Pair-end)<br>Su_Leaf_CK(Pair-end)<br>Su_3hRoot_CK(Pair-end)	Xueyan Zhang et al. 2013
Pub_Fiber_10dpa(Single-end)<br>Pub_Fiber_20dpa(Single-end)	Cotton Functional Genomics project, 2004
Pub_Leaf_CK(Single-end)	M-J Yoo et al. 2013
Pub_Seeds_10dpa(Single-end)<br>Pub_Seeds_20dpa(Single-end)<br>Pub_Seeds_30dpa(Single-end)<br>Pub_Seeds_40dpa(Single-end)	Simon Renny-Byfield et al. 2014
Pub_Seedling_5h(Single-end)<br>Pub_Seedling_15h(Single-end)<br>Pub_Seedling_30h(Single-end)	Tao Tao et al. 2013
**Detailed information of RNA-seq data of *G.hirsutum***	
calycle_1(single_end)<br>calycle_2(single_end)	SRR1695180
cotyledon_120h_1(single_end)<br>cotyledon_120h_2(single_end)	SRR1695167
fiber_25dpa_1(single_end)<br>fiber_25dpa_2(single_end)	SRR1695194
leaf_1(single_end)<br>leaf_2(single_end)	SRR1695175
ovule_35dpa_1(single_end)<br>ovule_35dpa_2(single_end)	SRR1695190

petal_1(single_end)<br>petal_2(single_end)	SRR1695177
pistil_1(single_end)<br>pistil_2(single_end)	SRR1695179
root_1(single_end)<br>root_2(single_end)	SRR1695173
seed_10h_1(single_end)<br>seed_10h_2(single_end)	SRR1695162
stamen_1(single_end)<br>stamen_2(single_end)	SRR1695178
stem_1(single_end)<br>stem_2(single_end)	SRR1695174
torus_1(single_end)<br>torus_2(single_end)	SRR1695176

Table S2. A list of PCR primer used in this study.

Target	Forward	Reverse
25S rRNA	CAGTACGAATACGAACCGTG	CAATGATAGGAAGAGCCGAC
Cotton_A_novel_05365	GGCATGAACAGTGGTGATTG	ATTCCCTTCTCCGTTGCTTT
Cotton_A_novel_13549	TCAAACCCACTGACCATTCA	GGTGGATGCTGATCGAAGAT
Cotton_A_novel_11640	CCTTCTCTGCAACTCGCTTC	GAGGCGCTATGTCTCCTTTG
Cotton_A_novel_00031	ACACGACGGGTTTCAAAGAA	CCATTTTCGAGCTCTCTTGG
Cotton_A_novel_00068	CAACCCAAAACCCAGAGTGT	TACCTCTTTGTTCCCCGTTG
Cotton_A_novel_07179	GCCAGAGTAGGCTCATCACC	ATCTTGCTCAGCGGTGCTAT
Cotton_A_29792	AGACACCACTTTGAAACCCG	CTGCTGAGGGTAGGTTTTGG

Table S3. Annotation table of all newly identified transcripts of cotton.
(in additional excel file)

Table S4. Annotation table of all retrieved lost genes.
(in additional excel file)

Table S5. GTF format of novel transcripts in *G. arboreum*.
(in additional gff file)

Table S6. GTF format of novel transcripts in *G. hirsutum*.
(in additional gff file)
